# Errata

**Published:** 1888-11

**Authors:** 


Errata.—On page 192, fourth line from bottom, for ''fetal'' read ''fatal;'' p. 195, ninth paragraph, for ''former'' read ''latter.''

On page 211, second line from top, for ''nercobiotic,'' read ''necrobiotic.''

On page 222, second line from bottom, read ''Dr. W. A. Morris'' (and not Dr. J. W. McLaughlin), ''was elected President.''

